# POCUS for Thrombus: Emphasizing the Importance of Initial Point-of-Care Ultrasound in the Management of Pulmonary Thromboembolism

**DOI:** 10.7759/cureus.58272

**Published:** 2024-04-14

**Authors:** Menkeoma Laura Okoli, Poonam Rao, Siima Kavuma, Ravi Vijay Bulusu, Shafik Hanna-Moussa, Khashayar Vahdat

**Affiliations:** 1 Internal Medicine, CHRISTUS Health/Texas A&M College of Medicine, Longview, USA; 2 Cardiology, CHRISTUS Health/Texas A&M College of Medicine, Longview, USA

**Keywords:** point-of-care ultrasound, heparin-induced thrombocytopenia, pulmonary embolism, deep venous thrombosis, coronary artery disease

## Abstract

Pulmonary embolism (PE) constitutes a substantial health burden among individuals in the United States. It ranks as the third most common cause of cardiovascular death aside from stroke and myocardial infarction. Diagnostic errors are common with PE as patients can present with non-specific symptoms or could be completely asymptomatic with PE being an incidental finding. Diagnostic errors can result in missed or late diagnosis of PE, which, in turn, increases health care costs, morbidity, and mortality rates. Hence, early diagnosis is crucial. Computed tomography pulmonary angiography (CTPA) remains the gold standard in PE diagnosis, despite exposure to high doses of radiation. Point-of-care ultrasound (POCUS) is an underutilized, non-invasive technique that aids in the early diagnosis of PE and can safely reduce the radiation from CTPA in cases where contraindication exists. POCUS has been shown to have a high sensitivity and specificity for early diagnosis of PE.

## Introduction

Pulmonary embolism (PE) stands as the second most misdiagnosed disease in some parts of the world, including European countries, as suggested by recent studies [1-6]. Data from these studies indicate that PE affects about one in four patients in the emergency department and over 49% of hospitalized patients [3]. However, in the United States (US), misdiagnosis of PE is relatively rare as cases of overdiagnosis with computed tomography pulmonary angiography (CTPA) have been reported [6,7]. PE constitutes a substantial health burden among individuals in the US as it is the third most common cause of cardiovascular death, aside from stroke and myocardial infarction [3-5,8-12].

Estimates of mortality caused by PE in the US stand at over 100,000 cases each year [9,11,12]. Given that the symptoms of PE are usually non-specific and can overlap with other diseases, diagnosis of PE can prove challenging [3,5,10-12]. Acute coronary syndrome, heart failure, and respiratory diseases represent the frequently diagnosed conditions in cases of patients with PE [3,4]. Implications of misdiagnosis include increased healthcare expenditure, [3] prolonged hospital course, and patient harm owing to adverse effects of unnecessary tests and medications [3].

Since the risk of missing a diagnosis of PE carries grave consequences and can result in mortality [8,13], it is imperative that physicians consider prompt and convenient modalities for PE diagnosis [4]. Point-of-care ultrasound (POCUS) has been shown to have a high specificity for PE diagnosis [13-15], more so for patients who have a contraindication to contrast CT or in critically ill patients [8,14]. Here, we highlight a case of a 78-year-old female who presented with non-specific symptoms and had a significant history of cardiovascular risk factors. She was initially managed as a case of acute coronary syndrome, but further investigation revealed evidence of sub-massive PE.

## Case presentation

A 78-year-old African American female, with a past medical history of stroke, type II diabetes mellitus, hyperlipidemia, hypertension, abnormal nuclear stress test, and colon cancer status post right partial colectomy a decade ago, with a reported normal colonoscopy three years ago, presented to the emergency department with complaints of generalized fatigue of a day duration. There was associated dizziness, near syncopal episodes, multiple episodes of diarrhea, nausea, and vomiting. She denied any other symptoms, including fever, chest pain, dyspnea, palpitations, lower extremity swelling, loss of consciousness, and abdominal pain. Physical examination was unremarkable. Vital signs on admission revealed an elevated heart rate of 106 bpm and a respiratory rate of 34 bpm. Blood pressure was 96/57, temperature of 36.7 ℃ (98.1 ℉), and oxygen saturation of 99% on room air. Venous blood gas obtained showed evidence of hypoxemia with normal CO_2_. Complete blood count (CBC) showed a normal white cell count, a low hemoglobin count of 10.9, and a reduced platelet count of 119,000. A comprehensive metabolic panel that was done showed evidence of acute kidney injury and metabolic acidosis.

**Figure 1:**
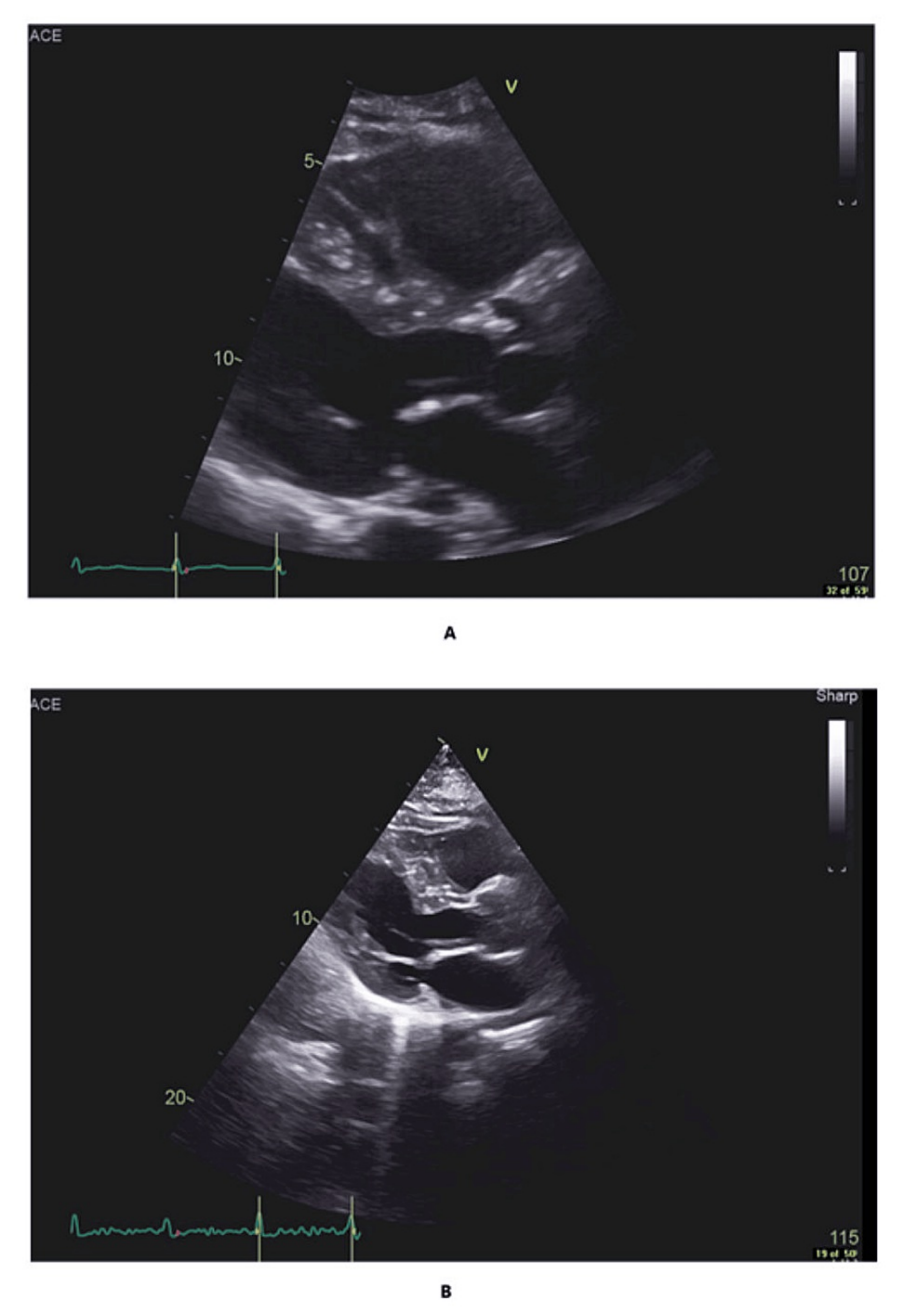
(A) Parasternal view of the transthoracic echocardiogram showing a dilated right ventricle suggestive of right ventricular strain. (B) Previous transthoracic echocardiogram on the chart showing a normal right ventricular pattern (for comparison purposes only).

**Figure 2:**
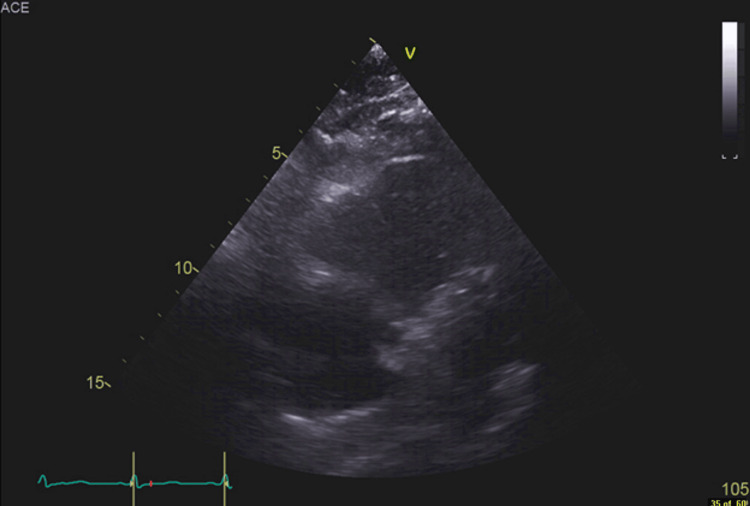
Apical view of the transthoracic echocardiogram showing a dilated right ventricle suggestive of right ventricular strain.

An electrocardiogram (EKG) showed ST depression of 0.5 mm in lead I, II, and V6. Cardiac troponin and lactate were elevated at 0.90 ng/mL and 3.3 mmol/L, respectively. Procalcitonin was elevated at 1.82 ng/mL, while urinalysis was unremarkable. Repeat troponin up-trended to 1.43 ng/mL. Chest x-ray showed no acute cardiopulmonary process. Given EKG findings, in addition to the up-trending troponin, a diagnosis of non-ST elevation myocardial infarction was made. She was started on heparin infusion, aspirin, and intravenous fluids. Upon further evaluation, D-dimer was found to be elevated at 50,282 ng/mL. Blood cultures were negative. She underwent cardiac catheterization, which revealed moderate two-vessel disease (left anterior descending and right coronary artery). Myocardial perfusion imaging showed non-hemodynamically significant disease, and medical management was recommended. A transthoracic echocardiogram revealed an ejection fraction of 60-65%, moderately dilated right ventricle with reduced right ventricular systolic function, interpreted as highly suggestive of pulmonary embolism with right ventricular strain (Figures 1A, 1B, 2). A ventilation and perfusion (V/Q) scan showed significant hypoperfusion throughout the right lung lobe.

**Figure 3:**
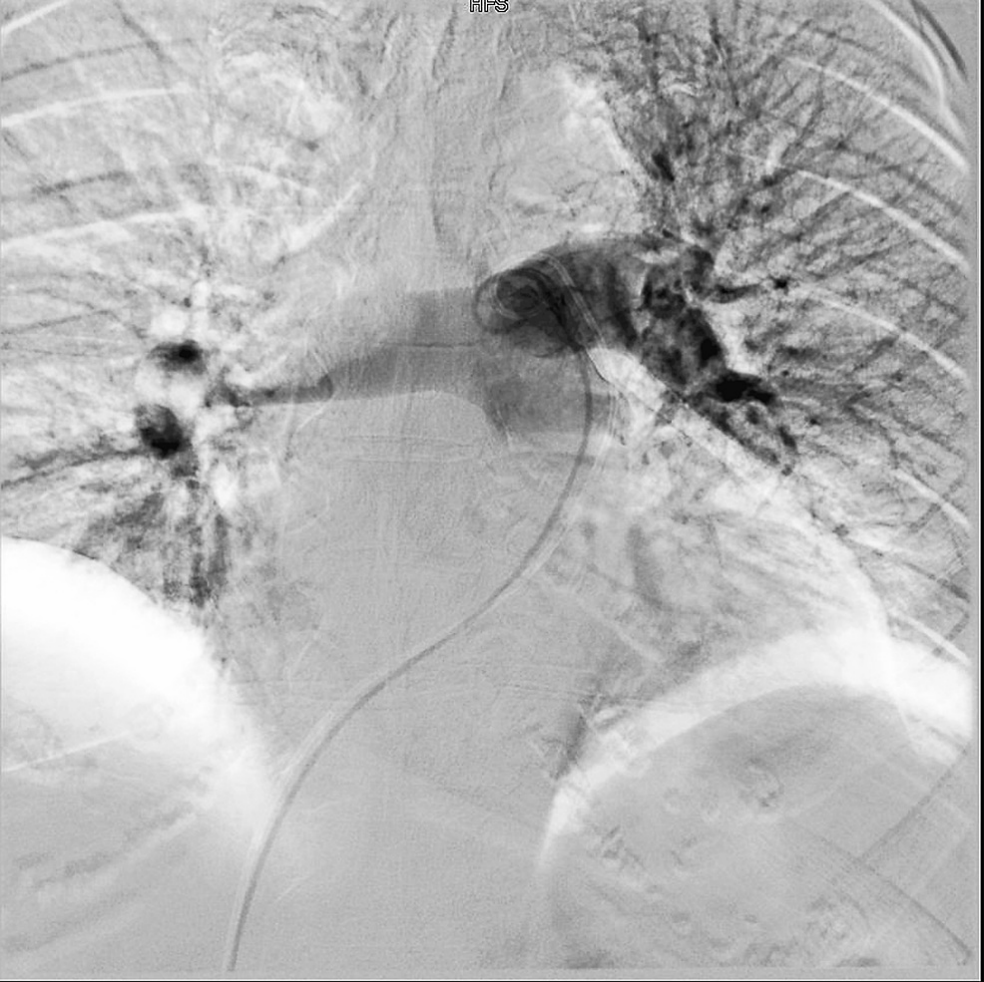
Pulmonary angiogram showing acute saddle pulmonary embolism (complete obliteration of the right pulmonary artery by thrombotic products with patent flow in the left pulmonary artery).

**Figure 4:**
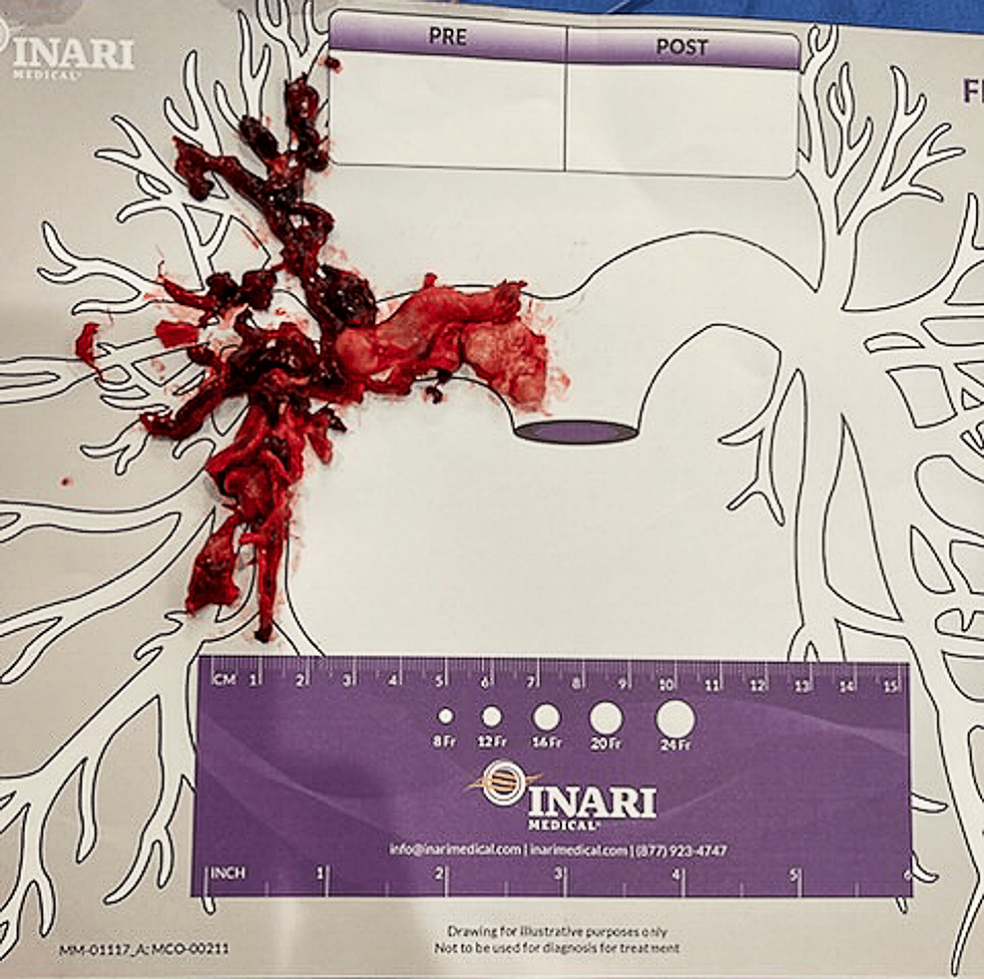
Blood clot retrieved from the right pulmonary artery via mechanical aspiration with the FlowTriever system.

**Figure 5:**
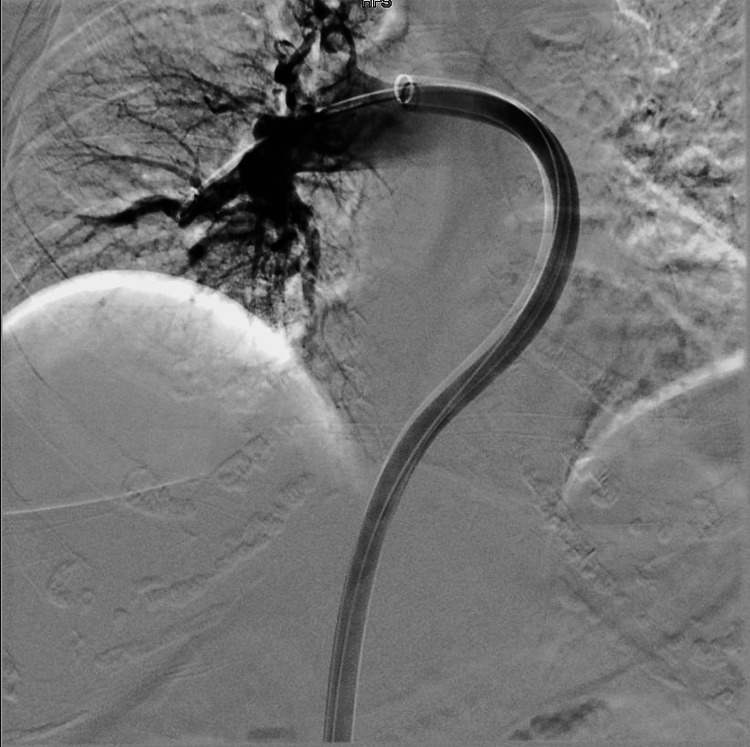
Pulmonary angiogram showing established blood flow into the upper, middle, and lower lobes of the pulmonary artery following a successful thrombectomy.

A duplex ultrasound of the bilateral lower extremity revealed acute deep vein thrombosis (DVT) of the left femoral, popliteal, and posterior tibial veins. Vitals taken at this time were normal, except for a BP of 154/74 and a heart rate of 105 bpm. A right heart catheterization pulmonary artery angiography with successful retrieval of the thrombus from the right pulmonary artery via mechanical aspiration with the FlowTriever system (Inari Medical, Irvine, CA) was performed (Figures 3-5).

Following the mechanical thrombectomy procedure, she developed a hematoma formation at the right inguinal venotomy site that resolved with pressure dressing. Heparin and aspirin were stopped. Repeat labs showed a low hemoglobin count of 6.5 gm/dL and a platelet count of 42,000. She received a unit of packed red blood cells (PRBC), platelet transfusion, and a dose of protamine sulfate and was then started on 10 mg of apixaban. Physical examination at this point was unremarkable, with the patient saturating 99% on room air. Normal fibrinogen, with elevated fibrin split products at 20 mcg/mL ruled out, disseminated intravascular coagulopathy. POCUS was done to evaluate the venous system, and it showed no evidence of bleeding or extravasation from the venotomy area.

Heparin platelet factor 4 antibody was positive at 2.48 optical density value above the reference range. A diagnosis of heparin-induced thrombocytopenia (HIT) was made, and she was switched to argatroban infusion. Platelet counts improved over the next few days, and she was started on warfarin bridge with argatroban infusion. Daily INR monitoring was commenced with an international normalized ratio (INR) goal of 4 as argatroban artificially prolongs INR measurements. On hospital day 14, she complained of right leg pain, which was relieved with pain medication. She denies any chest pain or shortness of breath. Right lower extremity non-pitting edema was observed with tenderness elicited on palpation. Lower extremity pulses were normal at this time. Examination of the right groin showed no swelling or tenderness. She was saturating 97% on room air. A repeat duplex scan of the right lower extremity showed a new extensive DVT throughout the right lower extremity despite being on continuous argatroban drip in addition to warfarin.

She was continued on argatroban-warfarin infusion bridging with a dose increase. The patient was advised that she is allergic to heparin and should not receive both heparin and heparin-related products. Life-long anticoagulation was recommended due to life-threatening thrombosis. Given the extensive clot burden and hypercoagulable state despite anticoagulation, transfer was initiated to a higher-level facility. Lupus anticoagulant test done was positive, raising suspicion for antiphospholipid syndrome. Upon revaluation the next day, the patient’s right leg appeared more swollen than the left side with no evidence of arterial compromise. However, she denied experiencing pain in the right leg. Cardiology planned popliteal access for right lower extremity DVT thrombectomy if she becomes symptomatic. She was eventually transferred to Baylor Scott & White Health the next day for continued management of her hypercoagulable state.

While at Baylor Scott & White Health, the initial workup showed negative antiphospholipid antibody syndrome - with negative repeat lupus anticoagulant test, anti-cardiolipin, and β2-glycoprotein antibodies. The serotonin release assay test was equivocal. Hence, treatment for HIT was continued. Serum protein with immunofixation detected no monoclonal peaks. CT abdomen and pelvis with contrast showed no findings of occult malignancy. Platelet count remained stable following transfer. She had a right lower extremity thrombectomy done on the fifth day of her hospital stay. Bridging with warfarin continued with maintenance of INR goal at 2-3. In addition, upper extremity venous duplex ultrasound done showed an acute occlusive DVT of the left brachial veins in the mid and distal upper arm, with superficial venous thrombosis of the left cephalic vein throughout the forearm. On the 10th day of hospitalization, the patient was discharged on a 3 mg daily dose of warfarin, and arrangements were made for the patient to follow up with a hematologist at the CHRISTUS Health facility. Repeat CBC showed that platelet counts had normalized, with levels of 243,000 at the time of discharge. The patient was discharged from the hospital with home health.

## Discussion

PE has been reported to be the most common misdiagnosed disease due to the non-specific nature of clinical symptoms and signs at presentation [1-3,8,10-12]. The patient described above presented with non-specific symptoms, including generalized fatigue, dizziness, near syncope, nausea, and vomiting. She had normal oxygen saturation on room air. The CTA PE protocol was indicated in her case, especially given symptoms of near syncope with up-trending troponin. However, this was not done due to significantly elevated creatinine on presentation. A V/Q scan, a preferred alternative, was also not done at the initial patient encounter. As discussed in other studies, the feasibility of performing a V/Q scan in emergent situations is limited due to multisystem factors, including the need for multiple staff members [3,11]. The patient was admitted with a diagnosis of acute coronary syndrome and was started on heparin infusion. She had cardiac catheterization done, which showed moderate stenosis of the coronary arteries. In this case, it was the echocardiogram that was done 16 hours later post admission that revealed evidence of sub-massive PE, which was further confirmed by a V/Q scan.

POCUS represents a non-invasive imaging modality that can be used in emergent settings to either rule in or rule out PE [11,16]. This is done based on findings indicative of right heart strain such as right ventricular systolic dysfunction, McConnell’s sign (defined as “right ventricular free wall akinesis with sparing of the apex”) [16], and the D-sign (represents the “bowing or flattening of the intraventricular septum into the left ventricle”) [11,13,16]. POCUS offers the benefit of removing the risks of contrast allergy as with CTA or radiation exposure, as seen with the V/Q scan while decreasing the time to diagnosis [11,14,16]. Various studies have shown that POCUS is very precise in identifying PE cases, with a specificity of 96.7% [11-13,15-17]. In this case, if POCUS had been performed at the presentation, the need for a cardiac catheterization procedure would have been obviated.

Numerous explanations could have accounted for the initial misdirection in this case, including anchoring bias, hindsight bias, cognitive errors, and multisystem errors, all bordering on quickness in arriving at a diagnosis, failure of consideration of other differential diagnoses, and lack of timeliness in obtaining necessary tests and appropriate consultations [3,4]. However, this case report serves not to assign blame but to promote learning opportunities, foster continuous self-reflective growth, and enhance our practice as clinicians. Various interventions, such as adopting the use of a treatment checklist guide, routine feedback system, root-cause analysis along with consultation with other physicians, and “debiasing,” have been proposed as methods to help avoid misdiagnosis [3,4].

While this case reinforces the need for consistent use of POCUS for the diagnosis of thromboembolic conditions, it is necessary to highlight that POCUS has a wide range of applications in the management of various diseases. POCUS has been shown to be useful in the diagnosis of numerous disease conditions in almost any organ system of the human body [18,19]. These conditions range from abdominal pathologies, such as aortic aneurysm, appendicitis, small bowel obstruction, ascites, cholelithiasis, renal abscess, hydronephrosis, pyelonephritis, and nephrolithiasis [18,19]; heart and lung diseases, including PE, heart failure, pulmonary edema, and airway compromise [18,19]; and diseases of the skin and soft tissues, including cellulitis, infections, and abscess [18,19]. In addition, POCUS is used in the management of obstetrics cases, such as confirming pregnancy and in the detection of ectopic pregnancy and abortions [18,19]. POCUS also plays a role in vascular access procedures to prevent the risk of complications [19]. The scope of POCUS is not limited to diagnosis alone, but it is also useful for treatment monitoring in some cases, providing a quick assessment of patients to further guide therapy [19].

Given the numerous benefits that POCUS offers, including its cost-effectiveness, combined with its ability to allow for rapid and timely patient assessment in emergencies to guide therapy and reduce the risk of misdiagnosis, it is imperative that physicians consider POCUS as an indispensable armamentarium. Additionally, physicians should always utilize POCUS as an initial diagnostic tool when indicated for effective patient management. The incorporation of POCUS use into the residency program curriculum, training of healthcare providers, and emphasis on mandatory POCUS use by all healthcare providers by hospital administration are necessary steps in fostering evidence-informed healthcare practices.

## Conclusions

This case report reinforces the need for consistent use of POCUS, a relatively quick and cost-effective approach, in situations where delays might be encountered with other imaging modalities. Although POCUS is a frequently used imaging modality in our facility, this case was an exception to the norm. In this case, the patient’s diagnosis was delayed but was not missed. Prompt treatment was initiated once a definitive diagnosis was made, and the patient had a favorable outcome. We hope that this case serves as a vehicle for promoting evidence-based healthcare practices, as we continue to push for high-value care for our patients.
